# The economic impact of cancer diagnosis to individuals and their families: a systematic review

**DOI:** 10.1007/s00520-022-06913-x

**Published:** 2022-03-02

**Authors:** Aymen Alzehr, Claire Hulme, Anne Spencer, Sarah Morgan-Trimmer

**Affiliations:** 1 College of Medicine and Health, University of Exeter, Exeter, UK; 2 College of Medicine and Health, Institute of Health Research, University of Exeter, Exeter, UK

**Keywords:** Cancer, Cancer survivors, Economic impact, Family/caregivers

## Abstract

**Background:**

The effect of a cancer diagnosis is wide-ranging with the potential to affect income, employment and risk of poverty. The aim of this systematic review is to identify the economic impact of a cancer diagnosis for patients and their families/caregivers.

**Methods:**

The search covered peer-reviewed journals using MEDLINE, EMBASE, CINAHL, Cochrane Library, Epistemonikos and PsycINFO databases. Quality appraisal was undertaken using CASP tools. Monetary values were converted to US Dollars/2019 using a purchasing power parities (PPP) conversion factor. The review included articles up to and including January 2020, written in English language, for patients with cancer aged ≥ 18 years and focused on the costs up to 5 years following a cancer diagnosis.

**Results:**

The search was run in January 2020 and updated in November 2021. Of the 7973 articles identified, 18 met the inclusion criteria. Studies were undertaken in the USA, Ireland, Canada, Australia, France, UK, Malaysia, Pakistan, China and Sri Lanka. The majority were cohort studies. Twelve reported out-of-pocket costs (range US$16–US$2523/month per patient/caregiver) consisting of medical expenses (e.g. surgery, radiotherapy and chemotherapy) and non-medical expenses (e.g. travel, food and childcare). Fourteen studies reported patient/caregiver loss of income and lost productivity (range 14–57.8%).

**Conclusions:**

A high percentage of cancer patients and their families/caregivers experience out-of-pocket expenditure, loss of income and lost productivity. Future research is needed to observe the effects of continuing changes to healthcare policies and social protections on the economic burden among cancer patients and their families/caregivers.

## Introduction

The economic impact of cancer on individuals and their families/caregivers is a global phenomenon. While technological advances in cancer detection and treatment have improved survival rates [1, 2], they are associated with high costs to healthcare systems and patients and their families/caregivers [3–5]. Differences in healthcare systems (e.g. publicly/privately funded) and social support schemes (e.g. whether there is an unemployment compensation during sick leave) can affect the type and amount of economic impact [6]. It is well known that cancer patients and their families/caregivers can experience economic burden, even within a universal healthcare system [7, 8].

Research on out-of-pocket (OOP) costs has been conducted in high and low/middle-income economies. A recent review [9] found that in high-income countries with publicly funded healthcare systems, cancer patients and their caregivers faced OOP costs that range from US$15 to US$400 monthly in Canada and US$58 to US$438 monthly in Australia. Altice et al. [10] report that in the USA, patients receiving oncology treatment can experience OOP costs ranging between US$316 and US$741 per month, and these costs were more than 20% of their annual income.

Additionally, data from several studies suggest that cancer patients are often not able to maintain full-time employment, having to reduce working hours or to cease work in some cases [10–13]. Losing a job after a cancer diagnosis can lead to both short-term (e.g. paying for bills or food) and long-term (e.g. losing a house) economic impact [14, 15].

Previous reviews have examined the economic impact of a cancer diagnosis. A recent review found that in publicly funded healthcare, cancer patients and their families/caregivers experience OOP costs ranging from $17 to $506 per month and income loss ranged from 17.6 to 67.3% [16]. In the USA, which has private healthcare systems, cancer survivors were found to incur financial hardship including OOP costs, income loss and lost working days [10]. Almost half of cancer survivors reported financial distress [10]. Another recent review focussed on OOP costs, reporting that cancer patients and caregivers in the USA spent a higher proportion of their income on OOP costs than that seen in high development index countries with publicly funded healthcare [9]. The same review reported that cancer patients and caregivers spent a higher proportion of their income on OOP costs in low- and middle-income countries than in high-income countries [9]. Other reviews focus on the impact on employment status, reporting that both reduced income and change in employment status (e.g. reduction in work hours and retirement) have been associated with a cancer diagnosis [12, 14].

**Fig. 1:**
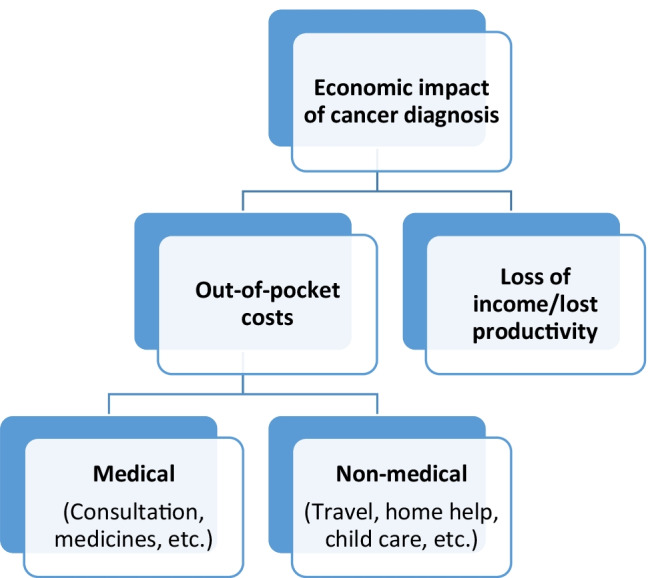
Economic impact on cancer diagnosis

While these previous reviews have considered the impact on patients and family/caregivers costs, they have not explored the types of costs and the key cost drivers [9, 10, 12, 14, 16]. This review aims to expand the evidence base by identifying the key cost drivers following a cancer diagnosis and the impact on individuals diagnosed with cancer and their families/caregivers. The review focusses on the 5 years following diagnosis given that previous evidence has shown that while costs are high immediately following diagnosis, they are also likely to be considerable 1–5 years after diagnosis [17]. Figure 1 illustrates the hypothesis with regard to the economic impact on cancer diagnosis.

## Methods

### Search strategy

The databases searched were MEDLINE, PsycINFO, CINAHL, EMBASE and Cochrane Library. Our review consists of three topics: (1) cancer, (2) cost and (3) patient/caregiver. Boolean operators and keywords were used with Medical Subject Headings (MeSH) when available (see Appendix). All search results were imported into EndNote X9 software, which was used to remove duplicates.

MeSH terms and keywords:

1. Cancer, oncology, chemotherapy, tumo?r*; MeSH Neoplasms.
2. (Financial adj (impact or toxicity or distress or burden or hardship or effect or difficult*)), (economic adj (burden or impact or implications hardship or difficult*)), friction cost, material hardship, societal cost, out-of-pocket, Labo?r market, deprivation, poverty, productivity loss, bankruptcy, catastrophic expenditure*; MeSH Cost of Illness.
3. Cancer survivors, carer*, famil*; MeSH Cancer survivors.

### Inclusion/exclusion criteria

Studies for this review had to be full‐text papers; published in peer-reviewed journals; published in English language; including individuals (aged 18 years or older) diagnosed with cancer; and focused on the cancer-related costs up to 5 years following diagnosis. Papers included randomised controlled trials (RCTs), quasi-experimental studies, cohort studies, case control studies, case studies, cross-sectional studies, longitudinal studies, systematic reviews, quantitative, qualitative and mixed methods studies. No restriction was applied to the type of cancer or setting.

Papers were excluded if they were editorials, commentaries, discussion or reviews, position papers and abstracts; focusing on the costs relating to pre-diagnosis; including costs accrued to sectors or systems rather than the individual and their family/caregivers; papers in which only total costs were reported (i.e. no breakdown of the components of the costs); and including participants under the age of 18 and childhood cancer survivors (see Table 1).

**Table 1:** Inclusion and exclusion criteria (PICOS)

Topic	Inclusion	Exclusion
Population	Cancer patients aged 18 years or older diagnosed with any type of cancer. No restriction was applied to the setting Up to five years from diagnosis	Cancer patients under the age of 18 and childhood cancer survivors
Intervention	No intervention was specified but over the time period this may include treatment, supportive care, diagnosis	None
Comparison	The focus is not on comparison, but where studies do include a comparator or control this may include treatment, supportive care, and diagnosis	None
Outcome	Cancer related costs up to five years from diagnosis including OOP medical and non-medical costs, loss of income and lost productivity accruing to the person diagnosed with cancer and their family/caregivers	(i) Costs relating to pre-diagnosis (ii) costs accrued to sectors or systems rather than the individual and their family/caregivers and (iii) Papers where only total costs are reported (i.e. no breakdown of the components of the costs)
Study	Full‐text papers in English language. Published in peer-reviewed journals Papers included randomised controlled trials (RCTs), quasi-experimental studies, cohort studies, case control studies, case studies, cross-sectional studies, longitudinal studies, systematic reviews, quantitative, qualitative and mixed methods studies	Editorials, commentaries, discussion or reviews, position papers and abstracts

### Screening

**Fig. 2:**
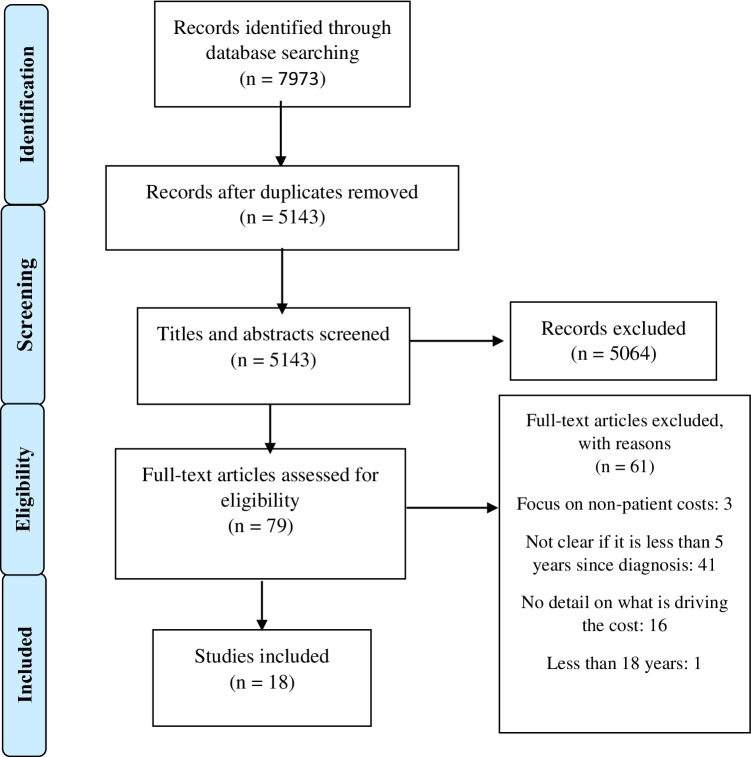
PRISMA diagram illustrating the study selection process

Initially, two reviewers screened the titles and abstracts. In the next stage, the full texts of the remaining studies were evaluated by two reviewers according to the pre-specified inclusion and exclusion criteria. We followed the Preferred Reporting Items for Systematic Reviews and Meta-Analyses (PRISMA) guidelines [18] (see Fig. 2).

### Data extraction

One reviewer extracted data from eligible papers into a bespoke data extraction form, which was checked for accuracy by a second reviewer. The extracted data included authors, year, country, objectives, cancer type, sample description (i.e. sample size, gender and age), study design, patients and/or caregivers perspective, time since diagnosis, costs timeframe, key findings, OOP cost per month (US$2019) (see Table 2).

**Table 2:** Summary characteristics and key findings of included studies

Authors/Year/Country/Objectives	Cancer type/Sample characteristics	Study design	Time since diagnosis	Patients and/or caregivers perspective	Measures	Costs timeframe	Key findings	OOP cost per month (US$2019)
Amarasinghe et al. (2019) [21] Sri Lanka Objectives: To estimate costs of managing patients with oral cancer	Oral cancer n = 69 Male 87% and female 13% Participants aged 40–81	Descriptive cross-sectional study	12 month	Both perspectives	Household costs: direct and indirect costs	Costs were estimated from the stage of presentation for treatment to 1 year of follow-up	Mean household cost for stage II patient was US$518 The annual household costs for stage III or IV patient was US$480	$21.33 per month for stage II $18 per month for stage III or IV
Carey et al. (2012) [22] Australia Objectives: To examine the social and financial impacts associated with supporting haematological cancer survivors	Haematological cancer n = 182. Support persons male & female (The paper report only % of female which is 71%) Participants aged 18–80	Cross-sectional survey	Within the last 3 years	Caregivers perspective	Direct and indirect costs	Support persons were asked to indicate costs over the past month	Overall, 67 (46%) support persons reported having at least one personal expense and 91 (52%) experienced at least one financial impact Male support persons and support persons of survivors in active treatment experienced more costs than other support persons Older participants reported fewer financial consequences	N/A
Céilleachair et al. (2012) [23] Ireland Objectives: To investigate the economic costs of cancer in the context of patients’ emotions and how these both shaped the patient and family burden	Colorectal cancer n = 2 patients and 6 carers, male (n = 8) & female (n = 14) Participants aged 44–82	Qualitative interviews	Within the previous year	Both perspectives	OOP costs	N/A	Important OOP costs included: travel and parking; costs of procedures; increased household bills; and new clothing Cancer impacted on employed individuals’ ability to work and decreased their income. The opportunity cost of informal care for carers, especially after diagnosis, was a strong theme	N/A
Ceilleachair et al. (2017) [24] Ireland Objectives: To investigate OOP costs incurred by colorectal survivors	Colorectal cancer n = 497, male 62% and female 38% Participants aged < 70 and ≥ 70 years	Cohort study	6–30 months	Patients perspective	OOP costs	The first year post diagnosis	The average OOP cost was €1589. Mean OOP costs for stage III disease were significantly higher than for those with other disease stages. Those aged 70 + had lower mean OOP costs than those < 70 (€1160 versus €1948). Those employed at diagnosis had a slightly higher OOP costs than those who were not (€1963 versus €1367)	$165.5 per month
Gordon et al. (2007) [25] Australia Objectives: To identify and describe the direct and indirect economic losses to breast cancer survivors	Breast cancer n = 272, female Participants aged 20–75	Longitudinal study	18 months following diagnosis	Both perspectives	Direct and indirect costs	Survey were obtained at five time-points: 6, 9, 12, 15 and 18 months from the date of diagnosis	Economic costs related to breast cancer may continue to affect women 18 months after diagnosis. Lost income, health services costs, and lost unpaid work were the greatest sources of economic burden	N/A
Hanly et al. (2013) [26] Ireland Objectives: To estimate financial and time costs associated with informal care for colorectal cancer	Colorectal cancer n = 154, caregivers male 18.2% and female 81.8% Participants aged 21–83	Cohort study	6–30 months	Caregivers perspective	Hospital-based costs, domestic-based time costs, domestic-based OOP costs and travel costs	Costs were collected during two phases: up to 3 months post-diagnosis (diagnosis and treatment) and during the last 30 days before questionnaire completion (ongoing care)	In the diagnosis and treatment phase, weekly informal care costs per person were: hospital-based costs, incurred by 99% of carers (mean = €393); domestic-based time costs, incurred by 85% (mean = €609); and domestic-based OOP costs, incurred by 68% (mean = €69) Ongoing costs included domestic-based time costs incurred by 66% (mean = €66) and domestic-based OOP costs incurred by 52% (mean = €52) The approximate average first year informal care cost was €29,842, of which 85% was time costs, 13% OOP costs and 2% travel costs	$399 per month
Jagsi et al. (2014) [27] The USA Objectives: To evaluate the financial experiences of a racially and ethnically diverse cohort of long-term breast cancer survivors (17% African American, 40% Latina) identified through population-based registries	Breast cancer n = 1502, female Participants aged 22–79	Longitudinal cohort study	4 years after diagnosis	Patients perspective	Changes in work and OOP costs	2005 to 2007 (4 year)	Overall, 33% reported financial decline since diagnosis. The median OOP expenses were ≤ $2000; 17% of respondents reported spending > $5000 Of the respondents who worked at some time after diagnosis, 27% decreased work hours, 7% were denied job opportunities because of cancer	N/A
Lauzier et al. (2008) [28] Canada Objectives: To estimate the burden from wage losses for Canadian patients	Breast cancer n = 459, women Participants aged 23–71	Prospective cohort study	Over the first 12 months after diagnosis	Both perspectives	Wage losses	The interviews conducted 6, and 12 months after the start of definitive treatment	On average, working women lost 27% of their projected usual annual wages (median = 19%) after compensation received had been taken into account. Higher percentage of lost wages was associated with a lower level of education, living 50 km or more from the hospital, lower social support, having invasive disease, receipt of chemotherapy, self-employment, shorter tenure in the job, and part-time work	N/A
Lauzier et al. (2013) [29] Canada Objectives: To 1) describe the extent of OOP costs among women and their spouses during the first year after diagnosis. 2) Identify women at risk of experiencing higher levels of OOP costs. 3) Describe effects of both OOP costs and wage losses on the family’s financial situation during the same period	Breast cancer n = 829, women and 391 spouses Participants aged 23–88	Longitudinal study	During the first year after diagnosis	Both perspectives	OOP costs	1-month interview focused on OOP costs related to surgery, 6-month interview focused on OOP costs related to adjuvant treatments, and 12-month interview focused on OOP costs related to any late treatments (surgical or adjuvant) and on other types of costs	Median OOP costs were $1002 (Canadian dollars). Spouses’ median costs were $111, or 9% of couples’ total expenses. Higher OOP costs were associated with higher education, working at diagnosis, living > 50 km from the hospital, and having multiple types of adjuvant treatment. When considered simultaneously with wage losses, OOP costs were not associated with perceived deterioration in the family’s financial situation; rather, wage losses were the driving factor	$95.58 per month for patients $16.38 per month for Spouses
Li et al. (2013) [30] The USA Objectives: To estimate lost productivity and informal caregiving and associated costs among partner caregivers of localized prostate cancer patients	Prostate cancer n = 88 partner caregivers Participants aged 34–80	Longitudinal cohort study	Within 1 year after diagnosis	Caregivers perspective	Care time and changes in work	Mailed follow-up surveys to patients and caregivers were administered at 6 and 12 months	The average working hours decreased from 14.0 h/week to 10.9 h/week. The mean annual economic burden among partner caregivers was $6063	N/A
Mahmood et al. (2018) [31] Pakistan Objectives: To explore the cost burden (i.e. direct medical costs, direct non-medical costs and indirect non-medical costs) incurred by breast cancer patients and their families over diagnosis and treatment	Breast cancer n = 200, women Participants aged 18 +	Cohort study	3 months to 2 years since diagnosis	Both perspectives	Direct medical, direct non-medical, indirect non-medical costs	N/A	The study found that direct medical care (US$ 1262.18) is the largest cost, followed by direct non-medical (US$ 310.88) and indirect non-medical costs (US$ 273.38)	$131 per month
Marti et al. (2016) [32] The UK Objectives: To describe the economic burden of UK cancer survivorship for breast, colorectal and prostate cancer patients treated with curative intent, 1 year post‐diagnosis	Breast, colorectal and prostate cancer n = 298, breast (n = 136), colorectal (n = 83), prostate (n = 79) Male 45% & female 55% Participants aged 18 +	Cohort study	12–15 months post-diagnosis	Both perspectives	Patients’ OOP costs and costs of informal care	The previous three months	Patients’ OOP expenses (mean: $US40) [mean: £25] and the cost of informal care (mean: $US110) [mean: £70]	$40 per month
Pisu et al. (2011) [33] The USA Objectives: (1) To describe OOP costs among minority and Caucasian participants in the in the BCEI, Breast Cancer Education Intervention, a randomized clinical trial of psychoeducational quality of life interventions for breast cancer survivors. (2) To examine the OOP burden, as measured by the proportion of income spent OOP, between the two racial/ethnic groups	Breast cancer n = 261, women Participants aged 21–83	Cross‐sectional	Within 2 years since diagnosis	Patients perspective	OOP costs	Authors examined the monthly OOP costs	OOP costs averaged $316 per month since diagnosis. Direct medical costs were $281 and direct non-medical were $66	$316 per month
Tison et al. (2016) [34] France Objectives: To investigate whether the labour market mobility of a population of cancer survivors 2 years after diagnosis differed compared to the French general population by focusing on the differences between self-employed workers and salaried staff	Mixed cancers The first dataset included 3967 individuals The second dataset (control group) contained 8066 respondents The paper did not report the % of male and female Participants aged 18–82	Case control study	2 years after diagnosis	Patients perspective	Changes in work	N/A	Salaried employees and self-employed workers from the general population were more likely to remain employed 2 years after 2010 compared to salaried employees and self-employed workers who survived cancer Among those who were employed in 2010, 14% for both self-employed and salaried cancer survivors were not employed 2 years later	N/A
Su et al. (2018) [35] Malaysia Objectives: This study aimed, firstly, to assess the determinants of return to work, secondly, to explore the amount of annual wage loss, and finally, to discover the determinants of wage loss among breast cancer survivors	Breast cancer n = 256, women Participants aged 20–79	Cross-sectional study	Within 1 year after diagnosis	Patients perspective	Wage losses	The data was collected over a period of 12 months	There was a 21% loss of or reduction in mean income within 1 year after diagnosis. The main risk factors for reduced wages or wage loss were belonging to the age group of 40–59 years, being of Chinese or Indian ethnicity, having low educational status, and not returning to work	N/A
Vayr et al. (2020) [36] France Objectives: To assess the rate of work adjustments 1 year after the diagnosis in a population of female breast cancer survivors, in the context of the French system of social protection	Breast cancer n = 185, women Participants aged 18—65	Prospective study	1 year after the diagnosis	Patients perspective	Work changes	1 year after the diagnosis	One year after the diagnosis, among 185 breast cancer survivors, 78 (42.2%) patients were working. Among them, 13 patients did not interrupt their occupational activity and 65 returned to work after a period of sick leave	N/A
Zhang et al. (2017) [37] China Objectives: To calculate the total cost of lung cancer treatment for lung cancer survivors in China within five years from the date of diagnosis	Lung cancer n = 195, male (n = 122) 62.56% & female (n = 73) Participants aged 29–89	Cohort study	5 years following diagnosis	Both perspectives	Direct medical costs, direct non-medical costs and indirect costs	First year after diagnosis	The average economic burden was $43,336 per patient, of which the direct cost per capita was $42,540 (98.16%) and the indirect cost per capita was $795 (1.84%). Of the total direct medical costs, 35.66% was paid by the insurer and 9.84% was not covered by insurance. The economic burden in the first year following diagnosis was $30,277 per capita	$2,523 per month
Humphries, et al. (2020) [38] Canada Objectives: To evaluate the wage losses incurred by spouses of women with nonmetastatic breast cancer	Nonmetastatic breast cancer n = 279, male 269, female 4 and unknown 6 Participants aged ≥ 18 years	Prospective cohort study	6 months after the diagnosis	Caregivers perspective	Wage losses	1 and 6 months after the diagnosis	Overall, 78.5% experienced work absences Spouses were compensated for 66.3% of their salary on average during their absence. The median wage loss was (mean, $1820) (Canadian dollars)	N/A

*OOP*, out of pocket costs

### Data synthesis and quality

All studies were assessed using the Critical Appraisal Skill Programme (CASP) [19] tool by one reviewer, and checked by a second reviewer (see Table 3). Due to heterogeneity in the included studies, it was not appropriate to use meta-analysis techniques. Instead, a narrative synthesis was performed to synthesize study characteristics and key findings.

**Table 3:** Quality assessment of studies

Cohort studies																													
Study		Did the study address a clearly focused issue?		Was the cohort recruited in an acceptable way?		Was the exposure accurately measured to minimise bias?			Was the outcome accurately measured to minimize bias?			Have the authors identified all important confounding factors?			Have they taken account of the confounding factors in the design and/or analysis?		Was the follow up of subjects complete enough?	Was the follow up of subjects long enough?			How precise are the results? Do you believe the results?		Can the results be applied to the local population?		Do the results of this study fit with other available evidence?		What are the implications of this study for practice?		Total (out of 12)
Amarasinghe et al. (2019) [21]		√		√		√			√			√			√		√	√			√		√		?		√		11
Carey et al. (2012) [22]		√		√		X			√			√			√		√	√			√		√		√		√		11
Ceilleachair et al. (2017) [2324]		√		√		√			√			√			√		?	√			√		√		√		√		11
Gordon et al. (2007) [25]		√		√		√			√			?			√		√	√			√		√		√		√		11
Hanly et al. (2013) [26]		√		√		X			√			√			√		?	√			√		√		√		√		10
Jagsi et al. (2014) [27]		√		√		√			√			√			√		√	√			√		√		√		?		11
Lauzier et al. (2008) [28]		√		√		√			√			√			√		?	√			√		√		√		√		11
Lauzier et al. (2013) [29]		√		√		√			√			√			√		√	√			√		√		√		√		12
Li et al. (2013) [30]		√		√		√			√			?			√		√	√			√		√		√		√		11
Mahmood et al. (2018) [31]		√		√		X			√			√			√		√	√			√		√		√		√		11
Marti et al. (2016) [32]		√		√		√			√			√			?		√	√			√		√		√		√		11
Pisu, et al. (2011) [33]		√		√		√			√			√			√		√	√			√		√		√		√		12
Su et al. (2018)		√		?		√			√			√			√		√	√			√		√		√		√		11
Vayr et al. (2020) [36]		√		√		√			√			√			√		√	√			√		√		√		?		11
Zhang et al. (2017) [37]		√		√		X			√			√			√		√	√			√		√		√		√		11
Humphries, et al. (2020) [38]		√		√		√			√			√			√		√	√			√		√		√		?		11
**Qualitative study**																													
Study		Was there a clear statement of the aims of the research?		Is a qualitative methodology appropriate?			Was the research design appropriate to address the aims of the research?			Was the recruitment strategy appropriate to the aims of the research?			Was the data collected in a way that addressed the research issue?			Has the relationship between researcher and participants been adequately considered?			Have ethical issues been taken into consideration?			Was the data analysis sufficiently rigorous?		Is there a clear statement of findings?		How valuable is the research?		Total (out of 10)	
Céilleachair et al. (2012) [23]		√		√			√			√			√			?			√			√		√		√		9	
**Case control study**																													
Study	Did the study address a clearly focused issue?		Did the authors use an appropriate method to answer their question?		Were the cases recruited in an acceptable way?			Were the controls selected in an acceptable way?			Was the exposure accurately measured to minimise bias?			Aside from the experimental intervention, were the groups treated equally?			Have the authors taken account of the potential confounding factors in the design and/or in their analysis?			How precise was the estimate of the treatment effect?		Do you believe the results?		Can the results be applied to the local population?		Do the results of this study fit with other available evidence?		Total (out of 11)	
Tison et al. (2016) [34]	√		√		√			√			√			√			√			√		√		√		√		11	

*√*, Yes; *X*, No; *?*, Can’t tell

In the display of findings in this systematic review, the local currencies in the included studies are converted to US Dollars/2019 when possible and appropriate for OOP and travel costs. To enable comparisons, costs were divided by purchasing power parity (PPP) exchange rate from the World Bank [20] to convert all non‐USD costs to USD costs and were transformed to reflect monthly expenditure (annual OOP costs were divided by 12 to obtain a monthly estimate) (see Table 2).

## Results

The search was run initially in January 2020 and updated in November 2021. A total of 7973 articles were identified. After removing duplicates, 5143 papers were included in the screening of titles and abstracts. Seventy nine papers remained for full text review. Eighteen studies were eligible for inclusion in the review [21–38]. The screening procedure can be seen in Fig. 2. Summary characteristics and key findings of included studies are reported in Table 2.

### Description of the studies

Ten countries were represented across the 18 studies; the USA [27, 30, 33], Ireland [23, 24, 26], Canada [28, 29, 38], France [34, 36], Australia [22, 25], the UK [32], Malaysia [35], Pakistan [31], China [37] and Sri Lanka [21]. The most common cancer types reported were n = 9 breast [25, 27–29, 31, 33, 35, 36, 38], n = 3 colorectal [23, 24, 26], n = 1 prostate [30], n = 1 oral [21], n = 1 haematological [22], n = 1 lung [37], n = 1 study included three cancers (breast, colorectal and prostate) [32], and n = 1 study included eleven cancers [34]. Of the 18 studies, n = 1 was a qualitative study [23], and the remaining were quantitative. Of the quantitative studies, n = 12 were cohort [24–32, 36–38], n = 4 were cross-sectional [21, 22, 33, 35], and n = 1 was a case control study [34]. The most common categories of economic burden reported in the 18 studies were n = 12 OOP costs [21–27, 29, 31–33, 37], and n = 14 loss of income/lost productivity [22, 24–28, 30–32, 34–38]. Regarding the cost perspective, n = 6 studies reported economic impact from the patient’s perspective [24, 27, 33–36], n = 4 from the caregiver’s perspective [22, 26, 30, 38], and n = 8 from both perspectives [21, 23, 25, 28, 29, 31, 32, 37].

### Quality of studies

Overall, the quality of included studies was rated as high (see Table 3). All reported the objectives and specified the population samples. For those conducting surveys or interviews, n = 8 studies had high response rates (≥ 50%) [22, 25–29, 32, 35] and n = 1 a low response rate (< 50%) [24], although n = 9 did not report the response rates [21, 23, 30, 31, 33, 34, 36–38].

### Out-of-pocket costs

There was a great deal of heterogeneity in the way that OOP costs were reported across the studies. The two main categories of OOP costs were medical (e.g. surgery, chemotherapy and medications) and non-medical (e.g. travel for treatments and childcare) expenses (see Fig. 1). In the studies that reported both OOP medical and non-medical costs, the highest average monthly cost ($2,523) was observed in China [37], and the lowest average cost per month ($16) was in Canada [29]. In terms of those studies reporting OOP medical costs separately, the highest average cost per month ($281) was observed in the USA [33], and the lowest monthly cost ($12) was in the UK [32]. The main cost drivers for medical costs were n = 9 treatment and medications [21, 22, 24–26, 29, 31–33]. Of the studies which reported OOP non-medical costs, the highest average cost per month ($66) was in the USA [33], and the lowest average cost per month ($26) was in Pakistan [31]. The type of cancer in both studies was breast cancer. The main cost drivers for non-medical costs were n = 9 travel for treatment [21–24, 29, 31–33, 37] and n = 6 homecare (e.g. cleaning and gardening) [22, 24–26, 31, 33]. Travel costs related to cancer were either reported within OOP expenses [26, 31, 32, 37] or calculated as separate costs. Mahmood et al. [31] revealed that the average travel cost came to $ 297 per month. Ceilleachair et al. [24] found an average monthly total cost of $166.25. The lowest average cost was observed in the UK, at $8 per month [32].

To best understand the cost burden from OOP, it is useful to assess the proportion of income spent on OOP costs for cancer care, a study [29] found that OOP costs represented a median of 2.3% of a family’s annual income. Another study [33] reported that the percentage of OOP costs was about 31% of the monthly income.

OOP costs may disproportionally also affect different groups in society that vary in terms of their socioeconomic, demographic and clinical characteristics. Socioeconomic characteristics associated with OOP cost burden included lower incomes [30, 33], diagnosed with cancer while working [24, 29], high level of education [29, 31]. Demographic characteristics linked to high OOP costs included younger age, [24, 25, 32] and being of an ethnic minority [33]. Regarding clinical characteristics, being at an advanced cancer stage [24] and receiving adjuvant treatment [23, 24, 29, 33] were associated with OOP cost burden.

### Loss of income/lost productivity

Loss of income/lost productivity was reported in 14 studies and was measured as loss of income [22, 23, 25, 28, 29, 31, 35, 38], time taken off from work [22], reduction in work hours [23, 27, 28, 30], lost working days [31, 37, 38], being unemployed [22, 34, 36], unpaid work [25], return to work [28, 35, 36], time costs [26, 32] and retirement [23, 30, 34, 36].

In the studies reporting the absolute loss of income, one study [25] reported that loss of income declined over time with a median loss of $5078 (0–6 months) to a median of $1553 (13–18 months). Another study [35] found that there was a 21% loss of mean income among breast cancer survivors within 1 year of diagnosis, from $1404 per month to $1110 per month.

Of the studies that reported a percentage change in income or in employment status**,** Lauzier et al. [28] revealed that an average 27% of the annual wage of cancer patients was lost over the first 12 months following a diagnosis. Another study showed that cancer patients reported an average 21% loss of their mean income within 1 year of a diagnosis [35]. Also, Vayr et al. [36] stated that 57.8% of cancer patients reported that they were not in work a year post diagnosis, while 42.2% were working and among them, 83.3% returned to work after taking a sick leave. Two studies reported that self-employment was associated with negative economic consequences as a result of not being able to benefit from the social security system [28, 34].

The individual characteristics that are either associated with a high percentage loss of income or which affect the employment status of patients and their families/caregivers included low educational status [28, 34, 35], being of an ethnic minority [35], having an advanced cancer stage [35] and receiving chemotherapy [28].

### Cancer caregiving

This review also identified the economic impact on the carers of patients. The types of costs involved in caregiving were OOP costs, cost associated with impact on employment and care time costs. With regard to OOP costs, caregivers face various expenses including travel costs, medication, food and clothes [22, 26, 29]. In terms of the impact on a caregivers’ employment from providing cancer care, an Australian study found that 40% of caregivers needed to be absent from work, 29% experienced income loss as a result of their caregiving and 8.6% had to leave work or close their business [22]. Also, a Canadian study reported that the absenteeism from work for caregivers was 78.5% with a mean wage loss of $1529 after they were compensated for wage loss due to work absence [38]. Regarding care time costs, Hanly et al. [26] found that over the first 12 months, care time costs accounted for 85% of the total costs incurred by caregivers who provided care at any phase of the disease.

## Discussion

This review was undertaken to identify the economic impact following a cancer diagnosis for patients and their families/caregivers and the individual patient characteristics associated with the costs. Among the results, differences in the types of economic impact were found. The main categories of economic impact that contribute to an economic burden to cancer patients and their caregivers were OOP cost and loss of income/lost productivity. The economic impact of a cancer diagnosis sits within the wider context of the structure of the healthcare system (e.g. national health insurance and co-payment systems) and the social welfare system (e.g. short or long-term sick leave and an early or late disability pension). Thus, in this section, we discuss these contextual issues in greater detail to address the heterogeneity in the findings.

### Out-of-pocket costs

OOP expenses are the most common costs that cancer patients face [39]. They occur across different health system models, including where individuals have health insurance coverage [27, 33].

Of the 18 studies, fourteen examined OOP costs in countries with universal healthcare [21–26, 28, 29, 32, 34–38]. Comparisons of cancer costs were limited in these studies due to factors, including different cancer types, the stage of disease at diagnosis and whether patients were in active treatment or follow-up care. However, this review has indicated that even in countries that have systems to provide universal healthcare coverage, patients with cancer face OOP cost burden. Moreover, the economic impact on patients and their families/caregivers may still occur in high-income countries with publicly funded healthcare models. This finding is comparable to a recent review that found cancer patients in public healthcare systems experienced increased OOP expenses [39].

Even in countries that provide universal healthcare, individuals who choose to have health insurance can incur additional OOP expenses [40, 41]. In Australia — a country with universal health insurance and optional private insurance — cancer patients faced high medical costs and other hidden costs [25]. The influence of the Australian healthcare system on cancer expenses has also been discussed in recent research [42] where cancer patients who had private insurance experienced higher economic burdens than those who relied on government-funded hospitals as a result of high co-payments (e.g. hospital fees).

This review found that in countries with private healthcare systems — USA [33], cancer patients experience higher OOP costs per month than those in low-income countries — Pakistan and Sri Lanka [21, 31]. On the other hand, in low- and middle-income countries where patients rely mainly on OOP payments for cancer care, the economic consequences of a cancer diagnosis can be extreme and may form a barrier to accessing cancer care [43, 44]. In this review, one study from Pakistan found that medical expenses were the main factor in the total cost of illness for a patient and their family/caregivers, which was, on average, $1262 [31]. This is a significant expense in a country where the average monthly wage is $268.10 [45] and when approximately half of all Pakistanis live in poverty [46].

The OOP costs of cancer found in the studies include expenses beyond the medical costs; these included costs related to home care tasks, such as cleaning and gardening [25, 29, 31, 33], making necessary home modifications for ease of living [23, 24] and paying for telephone, electricity and heating bills [23, 28, 32]. These findings are similar to those from previous research that found that patients with cancer experience additional costs related to household support [47, 48] and household bills [49, 50]. Transport costs, parking fees, accommodation for overnight stays and meals [23, 26, 29, 31] also created an economic burden for patients and caregivers. Cancer patients may need to travel long distances to access care or treatment when health services are located far from a patient’s home and or when frequent trips are necessary [23, 26, 29, 31].

In the studies that included OOP costs, individual characteristics were associated with the nature or type of economic impact following a cancer diagnosis such as lower income [29, 31, 33], diagnosed with cancer while working [24, 26], younger age [24, 25, 32], being of an ethnic minority [33], being at an advanced cancer stage [24] and receiving adjuvant treatment [23, 24, 29, 33]. These findings are in general agreement with those documented in prior reviews [9, 51], where individual characteristics, including a lower income, a high level of education, a younger age and an advanced stage of disease were connected with high OOP costs.

### Loss of income/lost productivity

There are observed variations regarding the impact of a cancer diagnosis on income loss and lost productivity between countries. The extent of support provided by social security systems is likely a factor here; Lauzier et al. [28] documented that in Canada, working women lost on average 27% of their annual income after receiving compensation (i.e. the system of insurance that reimburses workers in cases of illness). Canadian workers with cancer can make use of different forms of compensation, which can include up to half of their salary. Private employer insurance can cover a higher percentage of their salary compared to government employment insurance. They could also use annual paid leave as salary compensation if they are incapable of performing their normal duties [28]. In addition, this review confirms that self-employment is associated with economic difficulties after a cancer diagnosis [28, 34]. Self-employed patients reported limited access to government insurance coverage, resulting in much higher losses [28, 34]. These results are consistent with findings from previous studies that found a relationship between self-employment and an increased economic burden [52].

The working regulations of return to work following a cancer diagnosis, which is influenced by social security and health insurance systems (such as paid sick leave), may contribute to the variations in employment and economic hardship between countries [14]. In this review, a study from France, where a social support system grants patients on sick leave, found that one year after a cancer diagnosis, 42.2% of patients were working and among them, 83.3% returned to work after taking a sick leave [36]. However, this study did not mention whether patients on sick leave receive their full salary or only a percentage. Also, in a study from Malaysia, 40.6% of cancer survivors returned to work after their diagnosis [35]. It is worth noting that cancer patients in Malaysia who work in the governmental sector can take up to 2 years of paid sick leave, while those working in private sectors receive only 2 months [35]. The findings of this review are supported by a Dutch study, which found that the proportion of cancer survivors who returned to work decreased as a result of a change in policy in 2004 regarding sickness absence compensation that increased permitted sick leave from one year to two years [53].

The economic impact of a cancer diagnosis on patients and their families/caregivers is associated with some individual characteristics, which result in a high percentage loss of income or which affect a patient’s employment status. These characteristics included lower level of education [28, 34, 35], being of an ethnic minority [35] having an advanced cancer stage [35] and receiving chemotherapy [28]. These results are consistent with previous reviews that have shown that those with a low educational level, who are older, who have advanced cancer or are of an ethnic minority are more likely to experience loss of income and a change in their employment status [12, 54].

### Cancer caregiving

A number of included studies suggest that caregivers face considerable OOP costs, including travel costs, medication, food and clothing [22, 26, 29], loss of income/lost productivity [22, 30, 31, 37, 38] and care time costs, which range from $110 per month [32] to $2641 per month [26]. These high time costs may explain the negative impact caregiving has on employment and are similar to findings of previous research. A US study found that caregivers experience an average OOP cost of $1243 over a 12-month period, which mainly comprised travel costs [55]. Moreover, another review reported that cancer caregivers are more likely to lose a high percentage of income or may stop working [56].

### Strengths

This systematic review has several strengths. Multiple databases were used, and extensive keywords were searched to identify articles related to the economic impact of a cancer diagnosis on individuals and their families/caregivers. The review was not limited to a specific setting and targeted studies that included affected individuals other than cancer patients, including their family, caregivers and spouses.

### Limitations

This review has some limitations, which should be noted. In the analysis stage, it was challenging to make comparisons between the included studies as they differed across a number of factors. The studies included several countries with different average ages, different health systems and social support systems and they studied mixed stages of disease or cancer types, different time durations following diagnosis and different costs of items within categories. With such variations, it was impossible to make a direct comparison across studies. Additionally, to convert the costs into a common currency, purchasing power parities (PPP) were used to adjust local currencies in the included studies to the equivalent value of the US dollar as of 2019 [20]. However, the valuation of healthcare may be slightly different from PPP values, which were originally used for the net effect of all goods and services. This issue might influence the cost comparisons between different countries due to the limitations of exact estimates.

## Conclusion

This review shows that in the case of a cancer diagnosis, a considerable amount of OOP expenditure is incurred by patients and their families/caregivers and this can cause economic hardship. The impact of loss of income/lost productivity vary, which is likely to be an artefact of differences in social security systems. The economic impact on patients and caregivers varies across countries based on the structure of the healthcare system. Less heterogeneity among studies and increased standardisation of measures would make cost comparisons easier. Future research is needed to observe the effects of continuing changes to healthcare policies and social security systems on the economic burden of a cancer diagnosis.

## Data Availability

This study did not generate any new data.

## Appendix

MeSH terms and keywords:

1. exp Neoplasms/
2. cancer.ti,ab.
3. oncology.ti,ab.
4. chemotherapy*.ti,ab
5. tumo?r*.ti,ab.
6. 1 or 2 or 3 or 4 or 5
7. "Cost of Illness"/
8. (financial adj (impact or toxicity or distress or burden or hardship or effect or difficult*)).ti,ab.
9. (economic adj (burden or impact or implications hardship or difficult*)).ti,ab.
10. friction cost.ti,ab.
11. material hardship.ti,ab.
12. societal cost.ti,ab.
13. out-of-pocket.ti,ab.
14. labo?r market.ti,ab.
15. deprivation.ti,ab.
16. poverty.ti,ab.
17. productivity loss.ti,ab.
18. bankruptcy.ti,ab.
19. catastrophic expenditure*.ti,ab.
20. 7 or 8 or 9 or 10 or 11 or 12 or 13 or 14 or 15 or 16 or 17 or 18 or 19
21. Cancer survivors/
22. cancer survivors.ti,ab.
23. carer*.ti,ab.
24. famil*.ti,ab.
25. 21 or 22 or 23
26. 6 and 20 and 24
